# A Rare Spontaneous Gastrobiliary Fistula

**DOI:** 10.5005/jp-journals-10018-1111

**Published:** 2014-07-28

**Authors:** Adam Chwiesko, Grazyna Jurkowska, Boguslaw Kedra, Bogna Okulczyk, Zbigniew Kamocki, Andrzej Dabrowski

**Affiliations:** 1 Department of Gastroenterology and Internal Medicine, Medical University of Biatystok, Biatystok, Poland; 2 2nd Department of General and Gastroenterological Surgery, Medical University of Biatystok, Biatystok, Poland

**Keywords:** Gastrobiliary fistula, Peptic ulcer disease, Gastric ulcer.

## Abstract

We report the case of a 69-year-old man with a spontaneous gastrobiliary fistula. Internal biliary fistulas are usually the result of longstanding, untreated choledocholithiasis, cholecystolithiasis, peptic ulcers or rarely neoplasia. This patient’s unspecific clinical picture led to a late diagnosis, which was made during surgery.

**How to cite this article:** Chwiesko A, Jurkowska G, Kedra B, Okulczyk B, Kamocki Z, Dabrowski A. A Rare Spontaneous Gastrobiliary Fistula. Euroasian J Hepato-Gastroenterol 2014;4(2):101-103.

## INTRODUCTION

Fistulas between the extrahepatic bile duct and different abdominal organs are rare. Generally, these fistulas can be divided into spontaneous or iatrogenic and internal or external. It is assumed that 75% to 90% of all extrahepatic bile duct fistulas are spontaneous and develop as a result of cholecystolithiasis or choledocholithiasis. These fistulas develop as a complication of peptic ulcer in approximately 6% of cases, from neoplasia of the involved organs in about 4% of cases, and from iatrogenic causes in 9% to 23% of cases.

The fistulas mostly arise in the small intestine (5075%), large intestine (0-20%), and other organs (5%), including the stomach, pancreas, kidneys, skin and bronchial tree.^1-7^ The first description of a spontaneous gastrobiliary fistula was published in 1965 and only a few cases have since been described.^6,8-10^ Here, we present a case report of a patient with a spontaneous gastrobiliary fistula. This rare complication of longstanding peptic ulcer disease was successfully treated by surgeons.

## CASE REPORT

A 69-year-old man, with a more than 30-year history of chronic obstructive pulmonary disease (COPD) and peptic ulcer disease (PUD), was admitted because of fever, cough and dyspnea on exertion. Increased levels of acute-phase proteins, D-dimer (7030 ng/ml), hypoxia, and hypercapnia were found. Pulmonary embolism was suspected, but the investigation was negative. After 5 days, upper gastrointestinal (GI) bleeding occurred; however, endoscopy did not reveal the source of bleeding. A deformation of the duodenal bulb was observed. Abdominal ultrasound (US) revealed the presence of a heterogeneous, restricted fluid-solid mass in the liver hilus, close to the body of the pancreas. The patient was treated with antibiotics, metronidazole, bronchodilators, diuretics, H2-blockers, fraxiparine and transfusion of 2 units of packed red blood cells.

The patient was transferred to the department of gastroenterology and internal medicine, 4 weeks after the first symptoms. He complained of dull, middle epigastric postprandial pain, loss of appetite, and an approximately 20 kg loss of weight within the last 5 to 8 weeks. On physical examination, sparse pulmonary rales, midepigastric pain and pleural edema were observed. Laboratory tests showed hypochromic anemia, thrombocytosis and elevated levels of acute-phase proteins (Table 1).

**Figs 1A and B:**
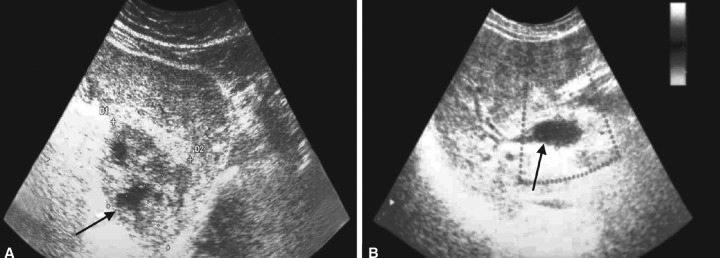
Abdominal ultrasound: (A) Barely separated hypoechoic lesion in the left hepatic lobe (arrow): 55.7 × 45 mm, with 2 echo-negative areas with diameters of 14 and 9 mm, with hyperechoic surroundings on the peripheral side of the left hepatic lobe; (B) the presence of pneumobilia in the left hepatic lobe. The common bile duct (CBD) dilated in the hilus (arrow) with a diameter of 14.9 mm and left lobular accentuation

**Fig. 2:**
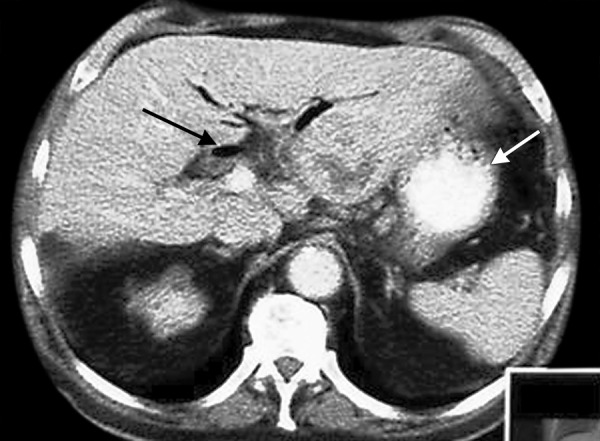
Abdominal contrast-enhanced computed tomography. In the left hepatic lobe (segment II), a heterogeneous, hypodense area is visible (white arrow), 50 × 55 × 35 mm, with tiny, partly connected liquid spaces. The dilated extrahepatic biliary ducts are indicated (black arrow). There is visible air inside the intrahepatic biliary ducts, CBD and gallbladder

The US revealed a hypoechoic lesion with hyper-echoic surroundings in the left hepatic lobe, which was suspected to be an abscess (Fig. 1A), and the presence of air in the extrahepatic and intrahepatic bile ducts (Fig. 1B). Abdominal computed tomography (CT) showed the presence of air in dilated intrahepatic bile ducts and the gallbladder, and the presence of a hypodense lesion in the left hepatic lobe (Fig. 2). During gastroscopy, a small amount of hemorrhagic erosion on the posterior wall in the lower part of gastric body, two ulcers (3 and 9 mm long), and a linear scar (12 mm long) on the anterior wall of the prepyloric region were found. The histological examination showed chronic gastritis without *Helicobacter pylori* infection. The clinical picture, imaging studies, and laboratory tests suggested a liver abscess and biliary duct fistula at the upper GI tract; thus, the patient was transferred to the department of surgery for further treatment.

**Fig. 3:**
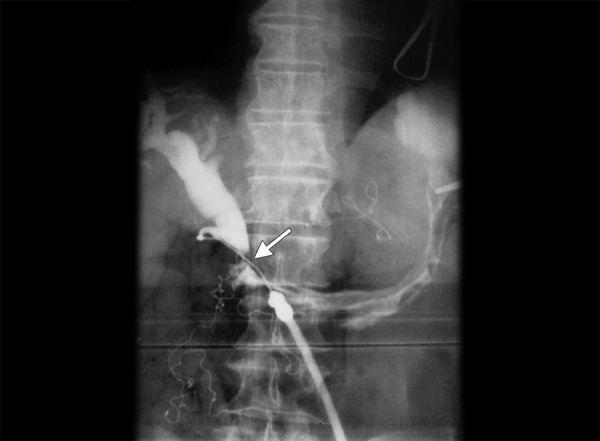
Intraoperative cholangiography after cholecystectomy. Dilatation of the extrahepatic biliary duct and a fistula (arrow) between CBD and stomach are visible. There is no passage of contrast agent into the duodenum

**Table 1:** **Table 1:** An overview of patient’s abnormal laboratory tests

*Results*			*Normal range*	
WBC: 8.63			4.0-10.0 × 10^3^/μl	
RBC: 3.83			4.5-6.0 × 10^6^/μl	
HGB: 11.2			14-18 gm/dl	
HCT: 35.4			40%-54	
MCV: 92.5			80-94 fl	
MCH: 29.3			27-34 pg	
MCHC: 31.7			31-37 gm/dl	
PLT: 355			130-350 × 10^3^/μl	
MPV: 8.0			7.0-12.0 fl	
CRP: 69.9			0-10 mg/l	
Fibrinogen: 650			200-400 mg	
ESR: 70			1-13 mm/h	

WBC: White blood cell; RBC: Red blood cell; HGB: Hemoglobin; HCT: Hematocrit; MCV: Mean corpuscular volume; MCH: Mean cell hemoglobin; MCHC: Mean corpuscular hemoglobin concentration; PLT: Platelets; MPV: Mean platelet volume; CRP: C-reactive protein; ESR: Erythrocyte sedimentation rate

During the operation, the surgeons found ‘an old’ wide pyloric ulcer perforation with a left subhepatic abscess and inflammatory infiltration of the hepatoduodenal ligament. After abscess evacuation, the cholecystectomy was performed. The intraoperative cholangiography showed proximal dilatation of the extrahepatic bile duct, no visible passage of contrast medium through the distal part of the common bile duct (CBD) into the duodenum, and the gastrobiliary fistula between the proximal CBD and stomach (Fig. 3). Then, the CBD was dissected and ligated in the distal part. Surgeons performed an antrectomy, combined with a truncal vagotomy and Billroth II gastrojejunostomy. The anastomosis between the dilated common hepatic duct and proximal jejunum was created. After 3 weeks, the patient was reoperated on because of leakage from the biliary anastomosis and intraperitoneal bleeding. The bleeding vessel was ligated and the anastomosis between the common hepatic duct and jejunum was repaired.

## DISCUSSION

Spontaneous, internal biliary fistulas are pathological communications between extrahepatic biliary ducts and abdominal organs. Most commonly, they develop because of longstanding, untreated choledocholithiasis, cholecystolithiasis, peptic ulcers or rarely as a result of neoplasia.^1,2,5,7,11^ In our patient, we assume the gastric ulcer was idiopathic due to the lack of *Helicobacter pylori* infection and non-steroidal anti-inflammatory drugs (NSAID); although, one can speculate that there was a possible *Helicobacter pylori* infection before antibiotics were given to treat an exacerbation of COPD. Unfortunately, there were no data in the patient’s files concerning this bacterial infection. We cannot exclude COPD as a risk factor of PUD in this patient.

The patients with gastrobiliary fistulas usually complain of postprandial right hypogastrium pain, epigastric pain and burning, elevated body temperature, nausea and postprandial vomiting. The presence of pneumobilia revealed on imaging is crucial for the final diagnosis in patients with a negative history of instrumental treatment (endoscopy or surgery of the biliary tree).^3^ The lack of hyperbilirubinemia is usually due to the patency of the gastrobiliary fistula as a primary path for biliary outflow.^10^ The diagnosis of biliary fistula is based on imaging studies and on endoscopy.^2,8-10,12^ An abdominal US reveals pneumobilia and extra- or intrahepatic dilation of the biliary ducts, which corresponds to cholestasis, cholelithiasis and choledocholithiasis. A CT scan may confirm pneumobilia and cholestasis, show the structure of adjacent organs, and reveal the presence of biliary fistulas, cholelithiasis and choledocholithiasis.

Uncomplicated gastrobiliary fistulas may be left untreated. Usually, these fistulas may heal spontaneously; however, pharmacotherapy is necessary for ulcer disease.^13-15^ Complicated gastrobiliary fistulas should be treated endoscopically or surgically.

Spontaneous gastrobiliary fistulas are serious, but rare complications of gastric ulcers. An unclear clinical picture results in a correct, but unfortunately late diagnosis of this disease, often in the course of surgery.
